# Acute Rheumatism Treated with Salicylic Acid

**Published:** 1877-08

**Authors:** Joseph D. Lomax

**Affiliations:** Medical Superintendent Marshall Infirmary, Troy, N. Y.


Acute Rheumatism Treated wilh Salicylic Acid.—*By Joseph D. Lomax, M.D., Medical Superintendent Marshall Infirmary, Troy, N. Y.*—The history of the following cases of acute rheumatism, treated in this hospital with salicylic acid, may be of interest to some of your readers. It includes all the cases of this disease that have been treated thus far with this remedy in the wards of the Marshall Infirmary.

I have reported the cases briefly, omitting to mention cardiac complications, and the effect of the medicine on the pulse and temperature, my principal object being to show the duration of the disease under this treatment, and the speedy relief which it brings to the patient.

The plan of treatment we employed was that adopted at the Bellevue Hospital, New York, as reported by Prof. Clark in the *Medical Record* for October 14th, 1876. The following is the prescription:

℞.—Salicylic Acid, . . . . . ʒiij

Bicarb. Soda, . . . . . . ʒij

Glycerine,

Water, . . . . . . aa. ℥ij. M.

Of this a tablespoonful is given every two hours for the first day, and afterward the same dose is given every six hours.

Case 1.—Male, aged 24, peddler, admitted November 18th, 1876. He had been ill two weeks; most of the joints of the lower extremities were red and swollen, and so painful that locomotion was almost impossible. Treatment was commenced at once, and the next day the joints were less painful, and could be bent with considerable ease. The patient continued to improve, and on the fifth day was up walking about the ward, feeling quite comfortable. On the seventh day treatment for rheumatism was suspended, as all the symptoms had subsided.

Case 2.—Male, aged 21, bar-tender, admitted December 11th, 1876. He had been ill three days; most of the joints of the extremities were affected, and those of the lower limbs so painful that walking was impossible. He was placed under treatment at once. The limbs could be moved with less dis comfort. On the 14th he was able to be up and walking about the ward. The patient continued to improve rapidly, and was discharged on the 18th, feeling well enough to go to work.

Case 3.—Male, aged thirty-one, grocer's clerk, admitted December 19th, 1876. He had been ill one week. The disease was confined mainly to the lower extremities, attacking the larger joints, which were swollen, and so painful that locomotion was impossible. Treatment was commenced at once, and in twenty-four hours the joints were less sensitive, and could be bent quite readily. On the third day of treatment he was able to walk across the ward. The patient continued to improve, and on the 24th asked to be discharged. He went to work in a few days after.

Case 4.—Female, aged about twenty, attendant in the insane department of this institution, admitted April 15th, 1877. She had had, for a week or two, more or less pain in her joints, but during the twenty-four hours preceding her admission she grew rapidly worse, and had become almost helpless; the disease had invaded not only many of the articulations of the limbs, but also many of those in the upper portion of the spinal column. She was put under treatment at once. The next day the progress of the disease seemed arrested, the joints were not so sensitive, and could be flexed with less discomfort. On the 19th, or fourth day of treatment, she was up, feeling quite free from pain in the joints and back. She continued to improve, and on the 22d returned to her duties in the asylum.

Case 5.—Male, aged 31, farm laborer, admitted May 22d, 1877. He had been ill three days, and many of the joints of the extremities had become more or less involved, rendering him unable to walk or feed himself. Treatment was commenced at once. The following day the pain was much relieved, and the limbs could be bent quite readily. He continued to improve, and on the 26th he was able, unassisted, to go down two flights of stairs to the lawn, and return. He was discharged, at his own request, on the 30th, feeling very well.

With the exception of Case 5, none of these patients complained of any unpleasant effects from the medicine. In two or three cases there were some deafness and ringing in the ears. In the female these symptoms were very marked, and this patient suffered for two or three weeks, after returning to work, from headache. She had never before been subject to headache.

To the cases reported above I will add the following:

The daughter of the reporter, aged 6 years. Symptoms of acute rheumatism were detected on the 13th of May last. The salicylic treatment was not begun until the following day, when many of the joints had become involved; in fact, the disease seemed to have invaded the articulations generally. Those in the upper portion of the spinal column were very sensitive, and when disturbed caused much distress. The little sufferer could not be moved without exciting much pain. The pulse was 140, and the temperature, taken in one of the axillæ, marked $101\frac{4}{5}°$. The first dose of medicine was given in the morning, 14th, and the same directions were observed in the treatment of this as were followed in the preceding cases, the dose being reduced to one teaspoonful. The next day, the 15th, the progress of the disease seemed arrested, the joints were less painful, and the patient could be moved more easily. The pulse had fallen to 128, and the temperature to $100\frac{1}{5}$. On the 16th the right elbow and left knee, also the back, were still very sensitive to motion, but the other affected articulations were less painful, and the patient could move herself a little. The pulse was 112, and the temperature 99. During the five succeeding days there seemed to be no further progress toward improvement, and the following change was then made in the prescription:

℞.—Salicylic Acid, . . . . . . ʒiij

Bicarb. Pot., . . . . . . . ʒj

Wine Colch. Sem., . . . . . ʒv

Glycerine,

Water, . . . . . . . aa ℥iv. M.

Of this two teaspoonfuls were given every six hours. This was continued for four days, but without any apparent beneficial results, and further experiments with salicylic acid were not made.

It is now seven weeks since the attack commenced, and the heart has been examined from time to time during the progress of the disease by several physicians, and no cardiac complication has yet been discovered. If this organ has escaped, an interesting question, it seems to me, might suggest itself, namely: whether this immunity is due to the particular kind of treatment adopted, or was this patient one of the favored five out of two hundred children attacked with acute rheumatism who escape heart complication? Such questions, of course, can be decided only by statistics, and hence I have reported this case.—*Med. and Surg. Reporter.*

